# Evaluating Amino Acid Profiles and Blood Gas Concentrations Between Single and Twin Merino Newborn Lambs

**DOI:** 10.1111/asj.70107

**Published:** 2025-09-10

**Authors:** Leesa‐Joy Dunstan, Michelle L. Hebart, Forbes D. Brien, Sue A. McCoard, Mariana Caetano

**Affiliations:** ^1^ Davies Livestock Research Centre, School of Animal and Veterinary Sciences The University of Adelaide Roseworthy South Australia Australia; ^2^ AgResearch Grasslands Palmerston North New Zealand

**Keywords:** baseline, neonatal, ruminants, sheep

## Abstract

As sheep production standards progress, and animals are bred for high production in terms of the number and weight of lambs weaned per ewe, research has identified a difference in the physiology of single lambs compared to multiple born lambs. The current study aimed to report the baseline amino acid (AA) profiles and blood gas concentrations in newborn, Merino single and twin lambs. From 120 days of gestation, 50 single‐bearing and 50 twin‐bearing, naturally mated Merino ewes were monitored for signs of approaching parturition. At birth, blood samples of the progeny were collected, and birth weight, rectal temperature, and meconium score were recorded. Blood plasma samples were analysed for AA profiles and blood gas concentrations were determined using an i‐Stat Alinity. Single‐born lambs had a higher birth weight (5.05 kg) compared to twins (4.24 kg; *p* < 0.05). Birth rank also affected rectal temperature and AAs aspartic acid, isoleucine, leucine, and phenylalanine, all being lower in twins compared to singles (*p* < 0.05). These baseline data provide insight into the physiological differences between single and twin lambs at birth from dams where there has been no treatment or intervention imposed.

## 1 Introduction

The Australian sheep industry is a significant contributor to the country's economy, through domestic and export earnings. The performance of the industry is driven by the number of lambs successfully reared and their individual contributions through wool and meat or milk (Rowe 2010). In New Zealand, producers have been particularly successful in increasing the number of lambs weaned per ewe (Flay et al. 2022), and as a consequence, the rate of multiple‐bearing ewes has also increased. Similar gains are observed in Australian systems (Haslin et al. 2023; Thompson et al. 2023). Multiple‐bearing ewes are a significant contributor to the overall flock productivity, provided the lambs survive long enough to contribute to productivity (Amer et al. 1999). There is still considerable scope for improving lamb survival in Australia, as productivity increases, but survival rates decrease with larger litter sizes (Hinch and Brien 2014). On average across Australia, mortality rates have been recorded as 10% in single, 15% in twin, and 33% in triplet born lambs (Thompson et al. 2023).

Lamb mortality is a complex trait, with multidisciplinary approaches required to understand causative factors including stock management strategies, maternal nutrition and health, and genetics. Baseline measurements are a crucial foundation in research trials, necessary for establishing reliable scientific conclusions. Animal trials have a need for accurate baseline or reference range values, as there is a large variation between breeds, age, and physiological categories, and even geographical regions or areas. Sheep are commonly used for research trials, for both animal and human research, and while there is extensive literature available on measurements such as amino acid (AA) profiles and blood gases, much is related to trials where a treatment or intervention has been applied. This makes it difficult to effectively understand the differences between single, twin, and triplet lambs regarding nutritional demands and physiological processes.

This preliminary trial aimed to quantify the blood gas and AA concentrations of newborn twin and single lambs. Baseline blood gas and AA profiles can be indicators of respiratory, metabolic, and nutritional health status. Understanding normal blood gas ranges can help detect conditions such as hypoxia, which is more likely to occur in twin lambs over singletons (Sales et al. 2018). Newborn lamb AA profiles provide insights into nutritional status and may reveal potential differences in physiological function such as metabolic disorders. Such data can then be utilized for subsequent research, as well as contribute to the existing values reported elsewhere. After establishing baselines or reference ranges, the data may inform research into potential interventions to improve multiple lamb survival.

## 2 Materials and Methods

This trial was approved by The University of Adelaide Animal Ethics Committee (Approval Number S‐2020‐061) and conducted in accordance with the Australian Code of Practice for the Care and Use of Animals for Scientific Purposes (2013).

## 3 Animals and Experimental Design

**FIGURE 1:**
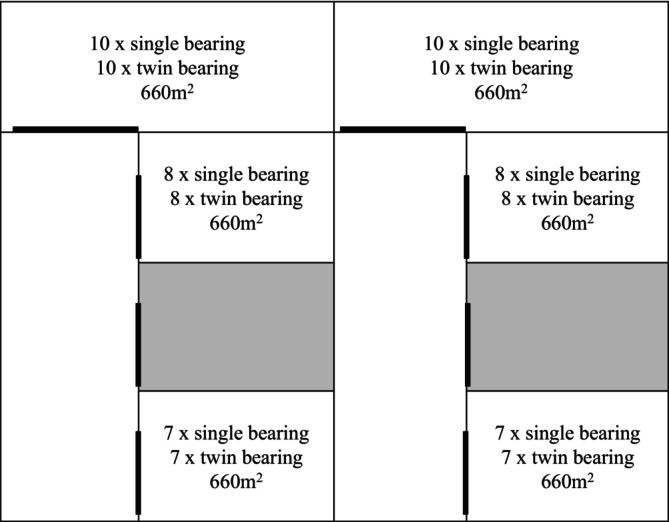
Pregnant ewe allocation pens. Pens located at The University of Adelaide, Roseworthy Campus, Roseworthy, South Australia, detailing the number of single or twin‐bearing ewes in each, including pen size (m^2^), laneways, and gates dictated by bold.

Sourced from The University of Adelaide, Roseworthy Campus Farm, extensively grazed flock, naturally mated to Merino rams, Merino ewes were confirmed pregnant via transabdominal ultrasonography. Fifty single‐bearing and 50 twin‐bearing ewes were selected based on pregnancy status (single‐ or twin‐bearing) and balanced for age (3 ± 1 years) and body condition score (BCS) (3.3 ± 0.5; 1–5 scale; Jefferies 1961). Ewes were randomly allocated into six pens, all with accessible shelters (Figure 1). Each pen was 660 m^2^ in size to ensure at least 10 m^2^ per ewe (Meat & Livestock Australia 2025). As ewes were intended to lamb in each pen, it was important to ensure sufficient space per ewe to avoid mismothering. Ewes were housed in each pen for lambing from approximately Day 120 of gestation. For the duration of the trial, animals were provided with clean fresh water and ad libitum oaten hay (Table 1). Ewes were monitored hourly for signs of lambing.

**TABLE 1:** Dry matter and nutrient composition of oaten hay offered for the duration of the trial.

Nutrient composition	
Dry matter (%)	90.60
ME (MJ/kg)	9.58
Crude protein (%)	8.70
ADF (%)	36.00
NDF (%)	55.20
Lignin (%)	5.18
Starch (%)	3.30
Crude fat (%)	1.60
Ash (%)	5.00
Calcium (%)	0.31
Phosphorus (%)	0.22
Magnesium (%)	0.16
Potassium (%)	1.00
Sulfur (%)	0.19

*Note:* Nutrition analysis performed by Forage Labs, Bendigo, Victoria, Australia, via wet chemistry.

## 4 Data Collection

Immediately after birth (within 30 min) and before the consumption of colostrum, each lamb (42 singles and 89 twins) was ear tagged for individual identification, and measurements were collected, including birth weight (kg), meconium score as a measure of stress at birth (4‐point scale; 0 = *no staining*, 1 = *mild staining*, 2 = *moderate staining*, and 3 = *severe staining*; Castro‐Nájera et al. 2006) and rectal temperature (°C). Blood was then collected via jugular venepuncture into lithium heparin tubes (Becton, Dickinson and Company “BD,” Franklin Lakes, New Jersey, USA). Lambs born unexpectedly as triplets were excluded from sampling (*n* = 9). Stillborn lambs, or lambs born unviable, were also excluded from analysis (two singles, four twins; total *n* = 6). Following the collection of samples, ewes and lambs were monitored for 24 h before returning to commercial management.

Whole blood of newborn lambs was analyzed for blood gas concentrations using an i‐Stat Alinity point‐of‐care analyzer and CG8+ cartridges (Abbott Point of Care, i‐Stat Alinity, Abbott Park, Illinois, USA) to measure blood pH, partial pressure of carbon dioxide (pCO_2_), partial pressure of oxygen (pO_2_), bicarbonate (HCO_3_), base excess (BE), saturated oxygen (sO_2_), total carbon dioxide (TCO_2_), sodium (Na), potassium (K), calcium (Ca), glucose (mmol/L), hematocrit (Hct; L/L), and hemoglobin (Hgb).

Bloods were then centrifuged (603.72 g/10 min at 20°C ambient temperature) and plasma aliquoted before being stored at −80°C for plasma AA analysis. A subset of plasma samples (10 single: 5 males and 5 females and 10 sets of twins: 9 males and 11 females; *n* = 30 total) was selected randomly from across all pens and analyzed for free AA profiles by the Australian Proteome Analysis Facility, Macquarie University (Macquarie Park, Sydney, New South Wales, Australia). Cystine could not be detected accurately in samples.

## 5 Statistical Analysis

A general linear mixed model (SPSS 28, IBM) was fitted to analyze the variation in birth weight, rectal temperature, meconium score, AA concentrations (μg/mL), and blood gases between singles and twins. Type of birth (single or twin), sex (female or male), and the interaction between type of birth and sex were fitted as fixed effects. Ewe ID, pen number (1–6), and birth period (a.m. or p.m.) were fitted as random effects to account for animals born to the same dam, different pens, and time of birth. A probability of *p* < 0.05 was considered statistically significant. The interaction between type of birth and sex was not significant, and therefore, values have not been reported.

## 6 Results

Sex had no effect on birth weight, rectal temperature, or meconium score (Table 2). However, twins had lower body weights at birth, being 0.78 kg less than single‐born lambs (*p* < 0.05). Twins also had a lower average rectal temperature, 0.86°C less than singles (*p* < 0.05). Meconium scores, on a scale of 0–3, were similar between litter sizes. Blood gas concentrations were not affected by birth rank or sex, with the exception of sO_2_ which was 18% lower in males than females (Table 3; *p* < 0.05).

**TABLE 2:** Birth weight (kg), rectal temperature (°C), and meconium score for type of birth and sex of newborn lambs. Values are presented as means ± SEM.

Variable	Type of birth				Sex	
	Twin	Single	*p*	Female	Male	*p*
Birth weight (kg)	4.24 ± 0.08	5.03 ± 0.10	< 0.001	4.61 ± 0.08	4.66 ± 0.091	0.631
Rectal temperature (°C)	37.84 ± 0.25	38.70 ± 0.32	0.034	38.19 ± 0.25	38.35 ± 0.28	0.659
Meconium score (0–3)	0.7 ± 0.12	0.8 ± 0.16	0.459	0.9 ± 0.12	0.7 ± 0.14	0.189

**TABLE 3:** Average blood gas concentrations for type of birth and sex. Values are presented as means ± SEM.

Variable	Type of birth				Sex	
	Twin	Single	*p*	Female	Male	*p*
pH	7.21 ± 0.02	7.23 ± 0.02	0.442	7.23 ± 0.02	7.22 ± 0.02	0.845
pCO_2_ (mmHg)	63.25 ± 2.74	62.11 ± 2.96	0.631	62.59 ± 1.45	62.76 ± 2.82	0.935
pO_2_ (mmHg)	24.34 ± 1.58	24.81 ± 2.01	0.853	26.40 ± 1.63	22.75 ± 1.75	0.104
HCO_3_ (mmol/L)	26.21 ± 1.17	26.13 ± 1.41	0.960	25.75 ± 1.23	26.58 ± 1.29	0.535
BE (mmol/L)	−2.25 ± 0.87	−1.51 ± 1.09	0.601	−7.73 ± 0.88	−2.02 ± 0.95	0.805
sO_2_ (%)	29.97 ± 2.46	33.09 ± 2.97	0.420	34.59 ± 2.38	28.47 ± 2.55	0.050
TCO_2_ (mmol/L)	27.44 ± 0.82	27.75 ± 0.95	0.749	27.74 ± 0.84	27.44 ± 0.87	0.712
Na (mmol/L)	146.92 ± 0.32	146.26 ± 0.37	0.193	146.56 ± 0.29	146.62 ± 0.31	0.855
K (mmol/L)	4.85 ± 0.08	4.69 ± 0.10	0.190	4.74 ± 0.08	4.80 ± 0.09	0.563
Ca (mmol/L)	1.36 ± 0.01	1.35 ± 0.02	0.479	1.35 ± 0.01	1.36 ± 0.01	0.352
Glucose (mmol/L)	4.06 ± 0.38	4.74 ± 0.54	0.305	4.58 ± 0.44	4.21 ± 0.47	0.545
Hct (L/L)	0.38 ± 0.01	0.36 ± 0.01	0.467	0.38 ± 0.01	0.36 ± 0.01	0.369
Hgb (g/L)	129.78 ± 3.94	125.64 ± 4.79	0.506	130.05 ± 3.86	125.38 ± 4.14	0.358
Ketones (mmol/L)	1.23 ± 0.56	0.63 ± 0.75	0.509	1.26 ± 0.61	0.58 ± 0.65	0.739

Concentrations of aspartic acid, isoleucine, leucine, and phenylalanine were all higher in the plasma of singles compared to twins (Table 4; *p* < 0.05). Sex had an effect on arginine and phenylalanine, where males had higher concentrations than females (*p* < 0.05). No other differences in AA concentrations between birth ranks or lamb sex were observed.

**TABLE 4:** Average plasma amino acid concentrations (μg/mL) for type of birth and sex. Values are presented as means ± SEM. Values are least‐square means ± SE.

Amino acid	Type of birth			Sex		
	Twin (*n* = 20)	Single (*n* = 10)	*p*	Female	Male	*p*
Alanine	44.14 ± 4.68	55.59 ± 5.45	0.124	45.39 ± 4.68	54.34 ± 4.51	0.131
Arginine	12.45 ± 1.42	11.25 ± 1.47	0.562	10.63 ± 1.15	13.07 ± 1.13	0.022
Aspartic acid	0.51 ± 0.07	0.76 ± 0.07	0.007	0.68 ± 0.07	0.59 ± 0.07	0.222
Asparagine	6.79 ± 0.98	9.42 ± 1.05	0.079	7.69 ± 8.44	8.52 ± 0.82	0.339
Glutamic acid	26.66 ± 1.39	28.05 ± 0.63	0.465	27.47 ± 1.39	27.24 ± 1.45	0.880
Glutamine	70.26 ± 6.61	81.12 ± 7.29	0.281	71.46 ± 6.11	79.91 ± 5.91	0.233
Glycine	68.15 ± 7.89	72.08 ± 8.11	0.619	66.58 ± 7.59	73.65 ± 7.62	0.252
Histidine	10.66 ± 1.13	12.96 ± 1.16	0.168	11.31 ± 0.91	12.33 ± 0.88	0.194
Isoleucine	5.11 ± 1.12	9.64 ± 1.15	0.009	7.09 ± 0.88	7.65 ± 0.86	0.435
Leucine	11.69 ± 2.39	20.92 ± 2.45	0.012	15.88 ± 0.85	16.73 ± 1.85	0.537
Lysine	11.44 ± 2.59	12.93 ± 2.85	0.702	13.33 ± 2.38	11.03 ± 2.31	0.402
Methionine	4.07 ± 0.89	5.70 ± 0.95	0.226	4.79 ± 0.75	4.98 ± 0.74	0.800
Phenylalanine	9.81 ± 1.05	12.36 ± 1.09	0.046	10.34 ± 0.92	11.84 ± 0.89	0.005
Proline	27.49 ± 2.34	31.26 ± 2.94	0.308	28.45 ± 2.57	30.31 ± 2.45	0.567
Serine	26.04 ± 2.85	25.03 ± 3.03	0.757	25.11 ± 2.79	25.96 ± 2.84	0.756
Threonine	21.16 ± 3.43	28.74 ± 3.59	0.139	24.02 ± 2.84	25.89 ± 2.77	0.484
Tryptophan	1.81 ± 0.37	1.90 ± 0.38	0.754	1.97 ± 0.36	1.74 ± 0.37	0.373
Tyrosine	13.74 ± 1.11	16.43 ± 1.19	0.111	14.81 ± 0.97	15.36 ± 0.95	0.592
Valine	39.47 ± 4.03	41.32 ± 4.24	0.756	41.03 ± 3.37	39.76 ± 3.29	0.699

## 7 Discussion

The results for blood gases and plasma AA concentrations reported in this study provide baseline values for newborn single and twin Merino lambs managed identically in a feedlot‐type management system with a mixed ration diet.

Twin lambs were lighter than their single counterparts at birth, consistent with previous studies in sheep (Hinch and Brien 2014; Lassala et al. 2011; Oldham et al. 2011; Bruce et al. 2021). Merino lamb survival, within the first 7 days, is optimized between 4.5 and 5 kg (Kirk et al. 2025). Lambs above the 4.5 to 5 kg range increase the risk of birthing difficulties and dystocia and thus the risk of lamb and ewe fatalities. This was previously reported by Refshauge et al. (2016) to be a serious problem in singles as twin and triplet lambs are often below 5 kg, although twins and triplets commonly suffer from birthing injuries, resulting in lamb fatalities, with the risk of death linear to birth weight. Interestingly, sex did not influence birth weight, which is unexpected as males are usually born heavier than females (Bruce et al. 2021; Sveinbjörnsson et al. 2021).

The higher concentrations of plasma sO_2_ in females versus males contrast previous studies (Dutra and Banchero 2011; Polglase et al. 2012) and may be indicative of differential lung maturation. In other animal models, such as humans and mice, it has been reported that sex has an effect on pulmonary surfactant production, in that female fetuses near full term reach lung maturation earlier than male counterparts (Nielsen 1985; Laube and Thome 2022). As surfactant helps to reduce the surface tension in the alveoli, earlier production would improve lung compliance and allow for increased gas exchange efficiency, thus increasing sO_2_ (Han and Mallampalli 2015). The average concentrations of sO_2_ in the current study were lower between male and female (28% vs. 35%) than the values reported by Silva et al. (2018) but within the range (0%–85%) as described by Dutra and Banchero (2011). The absence of any other differences in blood gases between birth ranks and between sexes indicates the lambs were not exhibiting metabolic acidosis or hypoxia. These results contrast the findings by Sales et al. (2018) where near‐term twin fetuses were reported to be hypoxic compared to singletons. The contrasting results may be due to the physiological stage of development (late gestation vs. newborn) or the level of maternal nutrition (undernourished vs. well nourished) in the study by Sales et al. (2018) compared to the present study. These results suggest that hypoxia and metabolic acidosis may be less of a risk for neonatal lambs when dams are not undernourished.

Late‐gestation twin fetuses from well‐nourished ewes, 1 week prior to term, have been reported to have lower plasma leucine, histidine, glutamine, and arginine concentrations, compared to singleton fetuses (van der Linden et al. 2013). These differences between birth ranks were not observed in the newborn lambs in the present study, which may be due to breed‐specific differences or, more likely, differences due to physiological stage. However, differences were seen between birth ranks in aspartic acid and isoleucine. Aspartic acid is a glucogenic AA, contributing to gluconeogenesis (Engelking 2015) while isoleucine plays a role in muscle protein synthesis (Litwack 2018), both expected to be higher in animals with higher affinities for energy and growth. This may explain the higher concentrations in single newborn lambs. Overall, the differences observed are small and may not be biologically relevant. AA concentrations, in both the maternal and fetal circulation, change drastically in the last 100 days of gestation and then again during parturition and birth (Kwon et al. 2003; McCoard 2016).

Arginine plays an important role in fetal development and growth (McCoard 2016), and phenylalanine is important in neonatal lambs by maintaining the production and function of neurotransmitters (Karahman et al. 2022). Elevated arginine concentrations in males compared to females, in the absence of an effect of birth weight, suggest that arginine may not be a key driver of fetal growth in males. This notion is consistent with the previous study conducted by McCoard (2016) where maternal arginine supplementation in mid‐late gestation increased the birth weight of females but not males. Arginine has an important role in male fertility through the production of nitric oxide and polyamines for spermiogenesis (Wu et al. 2009), suggesting that the elevated arginine in the plasma of males compared to females may be linked to testicular development. The biological implications of the elevated plasma levels of phenylalanine in males compared to females are unclear but may be associated with differences in sexual dimorphism of neurotransmitter production in sheep (Brown et al. 2011).

The results of this study contribute to providing baseline information on the metabolic state of male and female, twin, and single lambs at birth. The absence of major differences between birth ranks indicates similar metabolic states at birth and similar intrauterine experiences in both sexes. The most notable finding was the sex‐specific effect on sO_2_ which may indicate differential lung development. The data presented in the current study provides baseline values and reference ranges for plasma AA and blood gases that may inform future research addressing lamb mortality and morbidity.

## Conflicts of Interest

The authors declare no conflicts of interest.
